# Exploratory analysis of innate immune gene polymorphisms and COVID-19 in-hospital mortality in a large admixed Brazilian cohort

**DOI:** 10.3389/fmed.2026.1919908

**Published:** 2026-10-07

**Authors:** Mariene Ribeiro Amorim, Tania Regina Tozetto-Mendoza, Mariane Pereira Brito, Vanessa Cássia Raymundi, Rafaella Medina-Neves, Iara Moreno Linhares, Alvina Clara Felix, Layla Honorato, Noely Ferreira, Laura Sampaio Azevedo, Carlos R. R. Carvalho, Emanuelle Leonília Marques, Antonio Charlys Da-Costa, Maria Cassia Mendes Correa, Steven S. Witkin

**Affiliations:** 1 Faculty of Medicine, Institute of Tropical Medicine, University of São Paulo, São Paulo, Brazil; 2 Department of Obstetrics and Gynecology, Hospital das Clinicas da Faculdade de Medicina da Universidade de São Paulo, São Paulo, Brazil; 3 Faculty of Medicine, Heart Institute (InCor), University of São Paulo, São Paulo, Brazil; 4 Department of Obstetrics and Gynecology, Weill Cornell Medicine, New York, NY, United States

**Keywords:** ATG16L1, COVID-19, IFITM3, innate immunity, MBL-2, NLRP3, SARS-CoV-2, single-nucleotide polymorphisms

## Abstract

**Introduction:**

COVID-19 in-hospital mortality varies substantially among patients, suggesting a role for host genetic factors beyond established clinical risk predictors.

**Methods:**

This case-control study investigated the association of the candidate SNPs rs2241880 (*ATG16L1*), rs1800450 (*MBL2*), rs10754558 (*NLRP3*), and rs12252 (*IFITM3*), alongside clinical variables, with in-hospital mortality in consecutive hospitalized patients including 505 non-survivors and 535 survivors of acute COVID-19 treated at a public hospital in São Paulo, Brazil, between March and August 2020. Baseline demographic, clinical, and genetic characteristics were compared between groups, and independent predictors of mortality were identified using multivariate logistic regression with Firth's penalized likelihood as the primary model to address quasi-complete separation.

**Results:**

Deceased patients were significantly older, more frequently male, and had a higher comorbidity burden. In the Firth penalized model, male sex [exp(*β*) = 2.30; 95% CI: 1.33–4.02; *p* = 0.003], immunodeficiency [exp(*β*) = 5.06; 95% CI: 1.28–24.6; *p* = 0.019], and mechanical ventilation [exp(*β*) = 136; *p* < 0.001] were independent clinical predictors of mortality. Nominally significant exploratory associations were observed for the rs2241880 G/G genotype [*ATG16L1*; exp(*β*) = 2.27; 95% CI: 1.09–4.87; *p* = 0.028] and the rs12252 G/G genotype [*IFITM3*; exp(*β*) = 5.83; 95% CI: 1.02–45.7; *p* = 0.047] with increased mortality risk, and for the rs1800450 T/T genotype [*MBL2*; exp(*β*) = 0.03; 95% CI: 0.00–0.94; *p* = 0.047] with reduced mortality risk; however, none of these associations survived correction for multiple testing. No association was observed for rs10754558 (*NLRP3*). Variant allele frequencies differed significantly across ethnic groups, particularly for rs12252, whose G allele was more frequent in Black (21.6%) than in White (13.9%) patients (*p* < 0.001), and a sensitivity analysis adjusting for self-reported ethnicity confirmed the directional consistency of genetic associations with marginally attenuated effect sizes.

**Discussion:**

These exploratory findings suggest that innate immune pathways, including autophagy, antiviral restriction, and complement activation, may contribute to COVID-19 mortality risk in the highly admixed Brazilian population and underscore the importance of conducting genetic studies in populations currently underrepresented in global genomic databases. Replication in larger, multi-ancestry cohorts with matched clinical endpoints is warranted to confirm or refute these signals.

## 1 Introduction

Coronavirus disease 2019 (COVID-19), caused by the Severe Acute Respiratory Syndrome Coronavirus 2 (SARS-CoV-2), has emerged as one of the most devastating global health crises of the 21st century, with a high transmissibility that has spread to all five continents (1). While the majority of infected individuals experience mild or asymptomatic disease, a substantial proportion progress to severe illness, including acute respiratory distress syndrome (ARDS), multi-organ dysfunction, and death (2). Understanding the determinants of this clinical variability is essential for accurate risk stratification, optimal allocation of therapeutic resources, and the development of more effective preventive strategies.

Beyond well-established clinical and demographic risk factors—including advanced age, male sex, obesity, and comorbidities such as diabetes mellitus and cardiovascular disease (3, 4)—accumulating evidence indicates that single-nucleotide polymorphisms (SNPs) in immunoregulatory genes play a meaningful role in the host immune response to SARS-CoV-2. These genetic variations influence both susceptibility to infection and disease severity. Genome-wide association studies (GWAS) have been crucial in characterizing this genetic landscape. Early GWAS identified two major loci robustly associated with COVID-19 severity: the 3p21.31 region, encompassing genes including *LZTFL1*, *SLC6A20*, *FYCO1*, *CXCR6*, and *XCR1*, and the 9q34.2 locus containing the *ABO* blood group gene, with the risk allele at 3p21.31 conferring an odds ratio of 1.77 for respiratory failure (5). Subsequent large-scale efforts by the COVID-19 Host Genetics Initiative (HGI) expanded this landscape to 13 and subsequently 23 genome-wide significant loci across studies from 25 countries, implicating pathways such as antiviral restriction (OAS1/OAS2/OAS3), interferon signaling (IFNAR2, TYK2), and inflammasome activation (DPP9) (6, 7). More recently, analyses leveraging the full HGI Release 7 dataset identified up to 49 genome-wide significant loci (58). In parallel, candidate gene studies have demonstrated that polymorphisms in immune response genes, including *TLR7*, *TYK2*, *STAT1*, and *OAS1*, independently improve the prediction of COVID-19 severity beyond clinical variables alone, with specific genotypes associated with both increased and reduced risk of severe disease (8). Across these studies, marked inter-ethnic variability in the frequencies of risk alleles spanning HLA loci, cytokine genes, and viral entry receptors has been consistently reported, highlighting the need to investigate genetic determinants of COVID-19 in ethnically diverse populations (9). In light of this context, the present study focused on four candidate SNPs representative of distinct and biologically plausible innate immune pathways.

First, the *NLRP3* rs10754558 polymorphism has been previously linked to increased COVID-19 clinical severity (10). The NLRP3 protein is the rate-limiting component of inflammasome formation that drives the maturation and release of the pro-inflammatory cytokines IL-1β and IL-18, central mediators of the cytokine storm associated with critical disease (11). Second, autophagy constitutes an essential antiviral defense mechanism, and the *ATG16L1* gene codes for a protein essential for autophagosome formation. The rs2241880 variant is associated with reduced autophagic activity and increased SARS-CoV-2 susceptibility (12, 13). Third, the *MBL-2* gene encodes mannose-binding lectin (MBL), a key component of the complement lectin pathway. The rs1800450 polymorphism reduces serum MBL levels and impairs innate immune efficiency in virus opsonization and neutralization. This polymorphism has been associated with severe COVID-19 and increased risk of mortality (14, 15). Fourth, the interferon-mediated antiviral response depends critically on the product of the *IFITM3 gene*, a transmembrane restriction factor that prevents viral membrane fusion. The rs12252 polymorphism in this gene has been associated with impaired viral restriction and increased COVID-19 mortality across multiple populations (16, 17).

Given that the frequency of these polymorphisms varies significantly across ethnic groups, and considering the limited data on the evaluation of these genetic markers in the highly genetically diverse Brazilian population, this study aimed to exploratorily characterize their potential associations with COVID-19 in-hospital mortality. We conducted a retrospective cohort study involving 1,040 patients hospitalized with a confirmed diagnosis of COVID-19, comparing survivors and non-survivors, with the goal of generating hypotheses for future investigation in larger, multi-ancestry cohorts.

## 2 Material and methods

### 2.1 Study design and population

This case-control study was based on a consecutively enrolled cohort of acute COVID-19 patients admitted between March and August 2020 at Hospital das Clínicas da Faculdade de Medicina da Universidade de São Paulo (HCFMUSP), a public tertiary hospital in São Paulo, Brazil. All eligible patients with confirmed COVID-19 were included consecutively, with no selection based on clinical outcome. Nineteen patients were subsequently excluded due to insufficient DNA yield during sample processing, resulting in a final analytical cohort of 1,040 patients, 535 survivors (51.4%) and 505 non-survivors (48.6%) (18). All biological samples were stored at −80 °C until processed. The study period corresponded to the first wave of the COVID-19 pandemic in Brazil, during which no specific antiviral therapies were available in the public healthcare system. As documented by Pedroso et al. (19), HCFMUSP adopted a firm institutional position based exclusively on scientific evidence, establishing strict clinical protocols through its Crisis Management Committee and precluding the widespread use of unproven off-label agents (19). The predominant pharmacological intervention across both survivor and non-survivor groups was corticosteroid therapy, administered as a non-specific attenuator of severe inflammatory manifestations. Patient data, including demographic and clinical information, were extracted from electronic medical records. All data were anonymized prior to analysis. This study was approved by the National Research Ethics Commission of the Brazilian Ministry of Health (CONEP No. 6619485, CAAE: 75226023.7.0000.0068).

### 2.2 Candidate SNPs selection

Candidate SNP selection was based on a review of the published literature on host genetic determinants of COVID-19 severity and related respiratory viral infections, complemented by *in silico* functional annotation data from the Ensembl Variant Effect Predictor (VEP, GRCh38). SNPs were prioritized based on three criteria: 1) prior evidence of association with COVID-19 or related infectious disease outcomes in the published literature; 2) location in genes with established mechanistic relevance to innate immune pathways implicated in SARS-CoV-2 pathogenesis; and 3) functional annotation data supporting potential biological impact. The four selected variants for this study were the rs2241880 (*ATG16L1*), rs1800450 (*MBL2*), rs10754558 (*NLRP3*), and rs12252 (*IFITM3*), each represent a distinct and biologically plausible innate immune pathway: autophagy, complement lectin activation, inflammasome signaling, and interferon-mediated viral restriction, respectively.

### 2.3 Sample processing and genotyping

Genomic DNA extraction was performed using the QIAamp DNA Kit silica-column system (QIAGEN, Hilden, Germany). For surviving patients, DNA was extracted from buffy coat samples. For deceased patients, however, extraction was adapted to serum samples, as buffy coat was unavailable for this group. To mitigate the characteristically lower DNA concentration in serum compared to buffy coat and to ensure the highest possible yield, centrifugation at 800×*g* was performed to obtain a final serum volume of 200 μL, thereby securing the recovery of genomic DNA fragments suitable for genotyping. Following extraction, purification, and elution, the integrity and concentration of the extracted material were rigorously monitored, enabling standardization at a fixed concentration of 20 ng/μL, as quantified by NanoDrop 2000 spectrophotometry. Allelic discrimination genotyping was subsequently performed by real-time PCR using optimized TaqMan™ SNP Genotyping Assay (Thermo Fisher Scientific, Waltham, MA, USA) in combination with TaqPath™ ProAmp™ Master Mix (Thermo Fisher Scientific, Waltham, MA, USA), on a QuantStudio 5 thermocycler (LIM-52-FMUSP).

Genotyping of SNPs in the *ATG16L1*, *MBL-2*, *NLRP3*, and *IFITM3* genes was conducted by real-time PCR using TaqMan™ allelic discrimination technology. To ensure result reliability, positive controls for each genotype were established through initial screening runs, which served as reference standards for cluster formation in the allelic discrimination scatter plots. Reproducibility assays were additionally performed by processing samples in duplicate, confirming the consistency of genotypic calls and ensuring that the detected variants faithfully reflected the true genetic profile of each sample. Data analysis was completed using the ThermoFisher Connect online software, in which fluorescence signals captured at each amplification cycle enabled allelic discrimination. The inclusion of no-template controls and normalization of DNA input were critical steps to prevent non-specific amplification and to ensure that the calculated allele frequencies were representative of the study population. This rigorous approach effectively overcame the limitations inherent to the serum biological matrix, yielding reliable molecular data for downstream analysis.

DNA extraction was highly efficient, with a 100% (535/535) success rate for all buffy coat samples and 96.4% (505/524) for serum samples. To ensure homogeneity during allelic discrimination assays, reactions were grouped on test plates according to the nature of the biological material (serum- or buffy coat-derived DNA templates). Reproducibility was monitored by including homozygous and heterozygous controls in triplicate across all assay plates. Furthermore, non-template controls using nuclease-free water were employed to confirm the specificity and accuracy of the genotyping results.

### 2.4 Data and variables

Data collected included demographics (age, sex), clinical characteristics at admission (Body Mass Index, BMI), and pre-existing comorbidities, which were summarized using the Charlson Comorbidity score, as well as captured individually (e.g., hypertension, diabetes, obesity, cardiovascular disease, immunodeficiency, transplant recipient). Markers of acute disease severity, such as the need for Intensive Care Unit (ICU) admission, mechanical intubation, and total length of stay (LOS), were also recorded. The primary genetic data consisted of genotypes for four SNPs: rs2241880 (*ATG16L1*), rs1800450 (*MBL-2*), rs10754558 (*NLRP3*), and rs12252 (*IFITM3*). Missing values for continuous clinical covariates (age, BMI, Charlson score, and length of stay) were addressed through median imputation. Categorical comorbidities with missing entries were assumed to be absent (scored as 0).

### 2.5 Statistical analysis

Following the exclusion of patients with inconclusive genotyping or missing data for the primary outcome (in-hospital mortality), the resulting cohort (*N* = 1,040) was stratified by the main outcome (in-hospital mortality: “Survived” vs. “Deceased”). Baseline demographic, clinical, and genetic characteristics were compared between these two groups. Continuous variables (e.g., age) were presented as median and interquartile range (IQR) and compared using the Wilcoxon rank-sum test. Categorical variables (e.g., sex, comorbidities, SNPs) were presented as counts and percentages (%) and compared using the Pearson chi-square test or Fisher's exact test, as appropriate.

To identify independent predictors of in-hospital mortality, multivariate logistic regression models were constructed. Due to the presence of quasi-complete separation (e.g., nearly all deaths occurring in the ICU) and small sample sizes in certain genetic subgroups, Firth's penalized likelihood regression was used to obtain stable coefficient estimates and reliable *p*-values. Standard maximum likelihood logistic regression produces unreliable or non-convergent estimates in the presence of quasi-complete separation, a condition that occurs when one or more predictors almost perfectly predict the outcome. In our cohort, quasi-complete separation arose from the near-perfect association between mechanical ventilation and in-hospital mortality (98% of deceased patients required intubation vs. 43% of survivors), as well as from the small cell counts in rare genotype subgroups such as rs1800450 T/T (*n* = 9) and rs12252 G/G (*n* = 34). Under these conditions, standard logistic regression inflates coefficient estimates and widens confidence intervals to an unreliable degree. Firth's penalized likelihood method addresses this by introducing a penalty term into the estimation process that prevents regression coefficients from diverging to unreliable extreme values, yielding stable and interpretable estimates without requiring the exclusion of any predictor or observation. This approach has been specifically recommended for genetic association studies with rare genotypes and for clinical datasets with near-perfect predictors (20, 21).

The final model included age, sex, BMI, length of stay, ICU admission, intubation, hypertension, diabetes, obesity, and all four SNPs (rs2241880, rs1800450, rs10754558, rs12252). Results were expressed as Odds Ratios (OR), calculated by exponentiating the regression coefficients [exp(*beta*)]. In this context, an exp(beta) > 1 indicates an increased likelihood of mortality associated with the variable, whereas an exp(beta) < 1 indicates a protective effect. To facilitate the interpretation of the multivariate models, adjusted predictive probabilities (marginal effects) were calculated for each genotype. These values represent the expected percentage of survivors and deceased within each genetic group while holding clinical severity markers and demographics at their mean values.

Genotype and allele frequencies were determined by direct counting and then dividing by the number of chromosomes to obtain allele frequency and by the number of cases to obtain genotype frequency. Differences in allele frequencies between groups were assessed using the Pearson chi-square test. Ethnicity was self-declared by patients according to the Brazilian Institute of Geography and Statistics (IBGE) classification categories (www.ibge.com.br). Given the high degree of admixture characteristic of the Brazilian population, self-reported ethnicity may not fully reflect genetic ancestry.

As a sensitivity analysis, Bonferroni and Benjamini–Hochberg false discovery rate (FDR) corrections were applied across the eight genotype comparisons tested in the Firth model.

All statistical analyses were performed using R software v4.4.2 and associated packages, including *readxl*, *gtsummary*, *tidyverse*, *gt*, *logistf,* and *broom*. The packages *flextable* and *officer* were used for table construction. A two-sided *p*-value of <0.05 was considered statistically significant for all tests.

### 2.6 Targeted variant analysis using COVID-19 host genetics initiative data

To evaluate whether the four candidate SNPs showed any association signal in an independent large-scale dataset, a two-stage lookup analysis was performed using the publicly available summary statistics from the COVID-19 Host Genetics Initiative (HGI) Release 7, published in April 2022 (https://www.covid19hg.org/results/r7/). We confirmed that our cohort was not included in any HGI contributing study. First, the pre-filtered HGI R7 summary statistics files, which contain only variants reaching a suggestive significance threshold of *p* < 1 × 10^−5^, were searched for the four candidate SNPs: rs2241880 (*ATG16L1*), rs1800450 (*MBL-2*), rs10754558 (*NLRP3*), and rs12252 (*IFITM3*). None were identified in these filtered files. Then, we performed a complete regional association data (±500 kb around each candidate SNP position) were extracted directly from the full, non-filtered HGI R7 summary statistics files (GRCh38 build) using tabix v1.21, for three phenotypes: hospitalized COVID-19 vs. non-hospitalized COVID-19 (B1), very severe respiratory COVID-19 vs. general population (A2) and hospitalized COVID-19 vs. general population (B2). Additionally, proxy SNPs in high linkage disequilibrium (R^2^ ≥0.8) with each candidate variant were identified using the LDproxy tool (LDlink, NCI; https://ldlink.nci.nih.gov) (22), across three reference populations from the 1000 Genomes Project: European (EUR), African (AFR), and Admixed American (AMR, including Mexican, Peruvian, Colombian, and Puerto Rican populations); no Brazilian-specific reference population is currently available. Local Manhattan plots were generated using Python v3.12.2 *matplotlib* library.

## 3 Results

### 3.1 Baseline characteristics of the study cohort

This study analyzed data from a cohort of 1,040 patients hospitalized with confirmed COVID-19. Of these, 535 (51.4%) were survivors and 505 (48.6%) were non-survivors. The overall median age was 62 years (IQR: 50–70), and the cohort was predominantly male (620 patients, 60%). Compared to survivors, deceased patients were significantly older (median 66 vs. 57 years; *p* < 0.001) and more likely to be male (65% vs. 54%; *p* < 0.001). Median BMI at admission was similar between groups (27 kg/m^2^ in both) (Figure 1A). However, the distribution differed significantly (*p* < 0.001), with survivors presenting a slightly higher BMI, a pattern consistent with the obesity paradox previously reported in COVID-19 cohorts (23).

**Figure 1:**
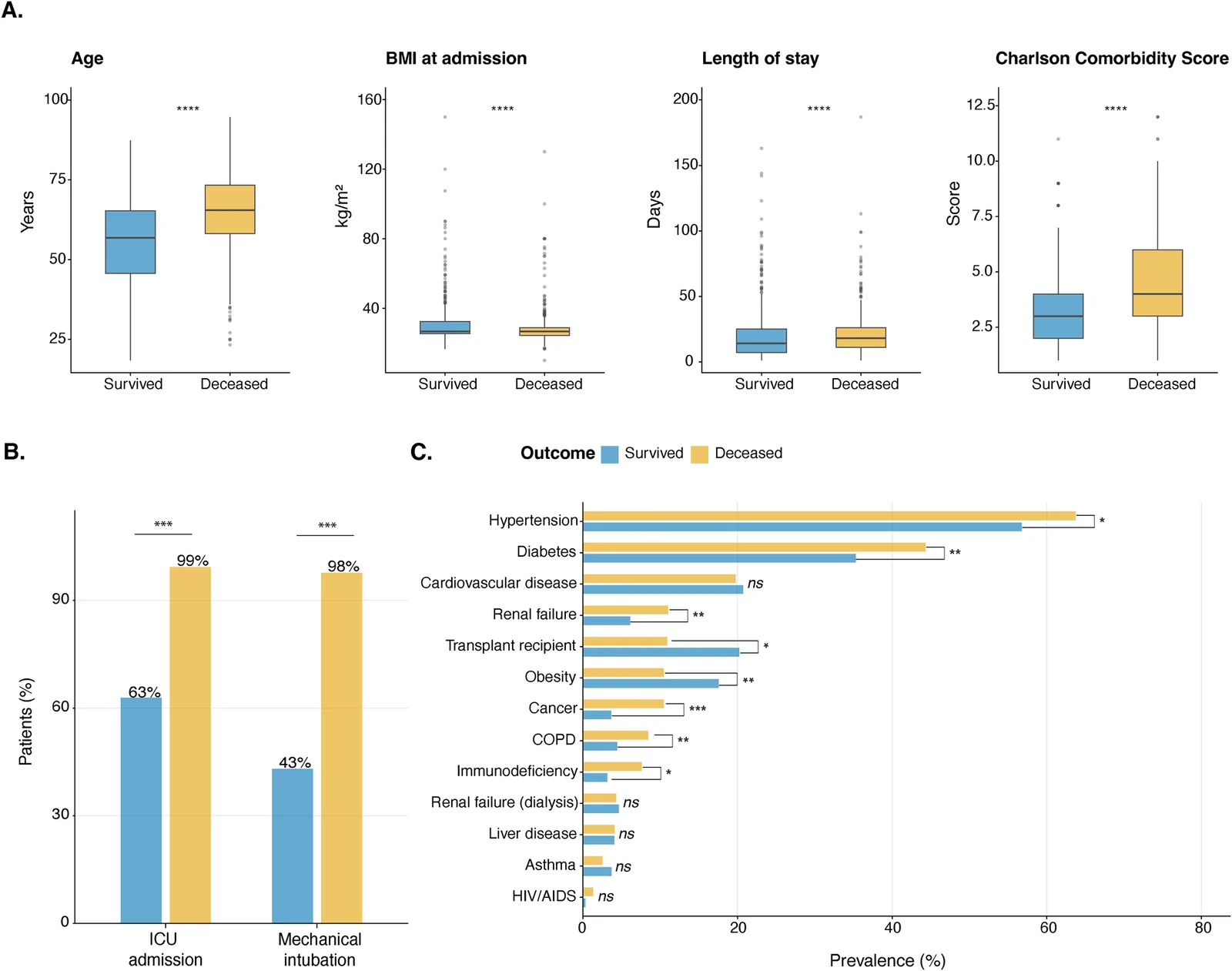
Clinical characteristics of hospitalized COVID-19 patients stratified by in-hospital mortality outcome. **(A)** Distribution of the continuous clinical variables age, BMI at admission, length of hospital stay, and Charlson Comorbidity Score, presented as boxplots with median and interquartile range. **(B)** Proportion of patients requiring ICU admission and mechanical intubation by outcome group. **(C)** Prevalence of pre-existing comorbidities among survivors and deceased patients. Significance brackets indicate results of Wilcoxon rank-sum tests **(A)** and Pearson chi-square or Fisher's exact test **(B,C)**. Percentages in panel **(C)** were calculated over patients with available data for each variable (complete-case analysis). *p < 0.05; **p < 0.01; ***p < 0.001; ****p < 0.0001.

Disease severity at admission was markedly higher among patients who died. ICU admission was required by 99% of deceased patients compared to 63% of survivors (*p* < 0.001), and mechanical ventilation was required by 98% vs. 43%, respectively (*p* < 0.001) (Figure 1B). Median length of hospital stay was longer in the deceased group (18 days, IQR: 11–26) than in survivors (14 days, IQR: 7–25; *p* < 0.001), reflecting the clinical trajectory of patients who deteriorated despite prolonged hospitalization. The burden of pre-existing comorbidities, as measured by the Charlson Comorbidity Score, was also significantly higher among deceased patients (median 4 vs. 3; *p* < 0.001) (Figure 1A).

Among individual comorbidities, hypertension (64% vs. 57%; *p* = 0.022), diabetes (44% vs. 35%; *p* = 0.003), renal failure (11% vs. 6.2%; *p* = 0.005), COPD (8.5% vs. 4.5%; *p* = 0.008), cancer (10% vs. 3.7%; *p* < 0.001), and immunodeficiency (7.7% vs. 3.2%; *p* = 0.048) were all significantly more prevalent among deceased patients. Notably, obesity was more prevalent among survivors (18% vs. 10%; *p* = 0.001), and transplant recipients also showed higher survival rates (20% vs. 11%; *p* = 0.030), though this latter finding should be interpreted with caution given the high proportion of missing data for this variable (73% of the cohort) (Figure 1C). Cardiovascular disease, asthma, liver disease, HIV/AIDS, and renal failure on dialysis did not differ significantly between groups. All comparisons are presented in Table 1.

**Table 1:** Baseline characteristics of hospitalized COVID-19 patients stratified by in-hospital mortality outcome (survived vs. deceased).

Characteristic	Total *N* = 1040^a^	Survived *N* = 535^a^	Deceased *N* = 505^a^	*p*-value^b^
Age (years)	61.5 (50.4, 69.6)	56.8 (45.6, 65.4)	65.5 (58.2, 73.3)	**<0** **.** **001**
Sex				**<0** **.** **001**
Female	420 (40%)	245 (46%)	175 (35%)	
Male	620 (60%)	290 (54%)	330 (65%)	
BMI at admission (kg/m^2^)	26.7 (24.2, 32.2)	28.4 (24.8, 34.2)	25.6 (23.4, 29.7)	**<0** **.** **001**
Missing, *n*	171	96	75	
Self-reported ethnicity				**0** **.** **012**
White	656 (65%)	354 (68%)	302 (61%)	
Black	81 (8.0%)	44 (8.5%)	37 (7.4%)	
Mixed	273 (27%)	120 (23%)	153 (31%)	
Asian	6 (0.6%)	1 (0.2%)	5 (1.0%)	
Missing, *n*	24	16	8	
Length of stay (days)	16.0 (9.0, 26.0)	14.0 (7.0, 25.0)	18.0 (11.0, 26.0)	**<0** **.** **001**
ICU admission	839 (81%)	337 (63%)	502 (99%)	**<0** **.** **001**
Mechanical intubation	722 (70%)	231 (43%)	491 (98%)	**<0** **.** **001**
Missing, *n*	3	0	3	
Charlson Comorbidity Score	3.0 (2.0, 5.0)	3.0 (2.0, 4.0)	4.0 (3.0, 6.0)	**<0** **.** **001**
Missing, *n*	27	16	11	
Hypertension	626 (60%)	304 (57%)	322 (64%)	**0** **.** **026**
Diabetes	413 (40%)	189 (35%)	224 (44%)	**0** **.** **004**
Obesity	147 (14%)	94 (18%)	53 (10%)	**0** **.** **001**
Missing, *n*	1	1	0	
Cardiovascular disease	211 (20%)	111 (21%)	100 (20%)	0.751
Missing, *n*	1	1	0	
Renal failure	89 (8.6%)	33 (6.2%)	56 (11%)	**0** **.** **006**
Immunodeficiency	26 (5.8%)	6 (3.2%)	20 (7.7%)	0.077
Missing, *n*	593	349	244	
COPD	67 (6.4%)	24 (4.5%)	43 (8.5%)	**0** **.** **012**
Missing, *n*	1	1	0	
Asthma	33 (3.2%)	20 (3.7%)	13 (2.6%)	0.372
Liver disease	43 (4.1%)	22 (4.1%)	21 (4.2%)	>0.999
Cancer	72 (7.1%)	19 (3.7%)	53 (10%)	**<0** **.** **001**
Missing, *n*	26	26	0	
HIV/AIDS	9 (0.9%)	2 (0.4%)	7 (1.4%)	0.154
Renal failure (dialysis)	47 (4.5%)	25 (4.7%)	22 (4.4%)	0.923
Transplant recipient	44 (15%)	28 (20%)	16 (11%)	**0** **.** **045**
Missing, *n*	756	397	359	
rs2241880 (ATG16L1)				0.543
A/A	363 (35%)	186 (35%)	177 (35%)	
A/G	490 (47%)	259 (48%)	231 (46%)	
G/G	187 (18%)	90 (17%)	97 (19%)	
rs1800450 (MBL-2)				0.143
C/C	765 (74%)	400 (75%)	365 (72%)	
C/T	266 (26%)	128 (24%)	138 (27%)	
T/T	9 (0.9%)	7 (1.3%)	2 (0.4%)	
rs10754558 (NLRP3)				0.465
C/C	456 (44%)	238 (44%)	218 (43%)	
C/G	452 (43%)	224 (42%)	228 (45%)	
G/G	132 (13%)	73 (14%)	59 (12%)	
rs12252 (IFITM3)				0.422
A/A	753 (72%)	396 (74%)	357 (71%)	
A/G	253 (24%)	124 (23%)	129 (26%)	
G/G	34 (3.3%)	15 (2.8%)	19 (3.8%)	

Bold values indicate statistical significance (*p* < 0.05).

a Median (IQR); *n* (%).

b Wilcoxon rank-sum test or Pearson's chi-squared test as appropriate. Complete-case analysis. Self-reported ethnicity coded as 999 (unknown) was treated as missing (*n* = 24).

Regarding the genetic characterization of the cohort, for rs2241880 (*ATG16L1*), the A/A, A/G, and G/G genotypes were observed in 35%, 47%, and 18% of the total cohort, respectively. For rs1800450 (*MBL-2*), the C/C genotype predominated (74%), with C/T in 26% and T/T in fewer than 1% of patients. The rs10754558 (*NLRP3*) showed a near-equal distribution between C/C (44%) and C/G (43%) genotypes, with G/G in 13%. For rs12252 (*IFITM3*), the A/A genotype was present in 72% of patients, A/G in 24%, and G/G in 3.3%. Genotype frequencies for all four SNPs did not differ significantly between survivors and deceased patients in the univariate analysis. The distribution of all genotypes by outcome is detailed in Table 1 and Figure 2.

**Figure 2:**
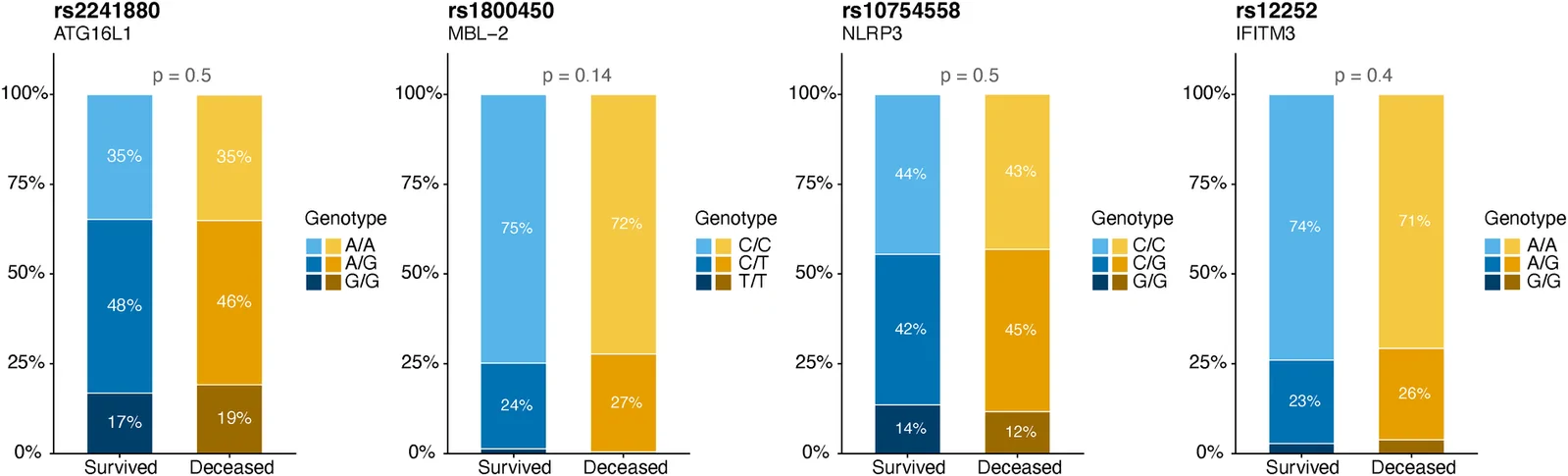
Genotype distribution of four candidate SNPs stratified by in-hospital mortality outcome. Stacked bar charts show the frequency of each genotype within the Survived (blue shades) and Deceased (orange shades) groups for rs2241880 (*ATG16L1*), rs1800450 (*MBL-2*), rs10754558 (*NLRP3*), and rs12252 (*IFITM3*). Within each outcome group, lighter shades represent the homozygous reference genotype, intermediate shades the heterozygous genotype, and darker shades the homozygous variant genotype. *p*-values were obtained from Pearson chi-square test or Fisher's exact test, as appropriate. No significant differences in genotype distribution were observed between groups in univariate analysis.

Allele frequencies were calculated for each SNP across the total cohort and stratified by outcome. The reference allele predominated for all four SNPs: rs2241880 (*ATG16L1*) A (58.5%), rs1800450 (*MBL-2*) C (86.4%), rs10754558 (*NLRP3*) C (65.6%), and rs12252 (*IFITM3*) A (84.6%). No significant differences in allele frequencies were observed between survivors and deceased patients for any of the four SNPs, consistent with the absence of significant genotype associations in the univariate analysis. Significant differences across ethnic groups were observed for rs2241880 (*ATG16L1*) (*p* = 0.001), rs1800450 (*MBL-2*) (*p* = 0.044), rs10754558 (*NLRP3*) (*p* = 0.019), and rs12252 (*IFITM3*) (*p* < 0.001); however, these findings should be interpreted with caution given the self-declared nature of ethnicity in this cohort, the high degree of admixture characteristic of the Brazilian population, and the very small size of the Asian subgroup (*n* = 6). Complete allele frequency data are presented in Supplementary Table S1 and Figure S1.

### 3.2 Multivariate predictors of in-hospital mortality

To identify independent predictors of in-hospital mortality, two complementary multivariate models were constructed. First, a standard Generalized Linear Model (GLM) was fitted, excluding acute severity markers (ICU admission and mechanical intubation), given that these variables act as mediators in the causal pathway between patient characteristics and death rather than as independent confounders. In this model, age (OR = 1.04, 95% CI: 1.01–1.06; *p* = 0.002), male sex (OR = 2.04, 95% CI: 1.32–3.18; *p* = 0.001), and length of stay (OR = 0.98, 95% CI: 0.97–1.00; *p* = 0.006) emerged as significant clinical predictors of mortality. Notably, the rs2241880 (*ATG16L1*) G/G genotype was also identified as a significant predictor in this model (OR = 1.87, 95% CI: 1.05–3.40; *p* = 0.036), providing an initial signal of genetic association independent of major clinical covariates in this cohort (Supplementary Table S2).

To obtain stable estimates in the presence of quasi-complete separation, where nearly all deaths occurred among patients admitted to the ICU and requiring mechanical ventilation, and to account for the small sample sizes of rare genotypes such as rs1800450 (*MBL-2*) T/T (*n* = 9) and rs12252 (*IFITM3*) G/G (*n* = 34), a second model was fitted using Firth's penalized likelihood regression. This approach is specifically designed to correct the bias introduced by separation and small cell counts that cause standard logistic regression to produce unreliable or non-convergent estimates. The full Firth model included age, sex, BMI, length of stay, ICU admission, mechanical intubation, major comorbidities, and all four SNPs (Table 2).

**Table 2:** Multivariate Firth penalized likelihood regression analysis of independent predictors of in-hospital mortality.

Characteristic	exp(Beta)	95% CI	*p*-value
Age	1.03	1.00, 1.06	**0** **.** **048**
Sex			
Female	—	—	
Male	2.30	1.33, 4.02	**0** **.** **003**
BMI at Admission	0.99	0.98, 1.01	0.5
Charlson Score	1.12	0.88, 1.43	0.4
Length of Stay	0.95	0.93, 0.96	**<0** **.** **001**
Requirement for ICU			
No	—	—	
Yes	4.43	0.26, 653	0.3
Intubation requirement			
No	—	—	
Yes	136	30.8, 1,232	**<0** **.** **001**
Hypertension			
No	—	—	
Yes	0.87	0.46, 1.63	0.7
Diabetes			
No	—	—	
Yes	1.04	0.55, 2.00	0.9
Obesity			
No	—	—	
Yes	0.57	0.27, 1.22	0.14
Renal failure			
No	—	—	
Yes	1.75	0.55, 6.17	0.4
Immunodeficiency			
No	—	—	
Yes	5.06	1.28, 24.6	**0.019**
COPD			
No	—	—	
Yes	0.41	0.12, 1.63	0.2
Cancer			
No	—	—	
Yes	1.27	0.31, 6.16	0.7
Genotype rs2241880			
A/A	—	—	
A/G	1.27	0.69, 2.34	0.4
G/G	2.27	1.09, 4.87	**0** **.** **028**
Genotype rs1800450			
C/C	—	—	
C/T	0.72	0.40, 1.32	0.3
T/T	0.03	0.00, 0.94	**0** **.** **047**
Genotype rs10754558			
C/C	—	—	
C/G	0.90	0.51, 1.59	0.7
G/G	0.73	0.31, 1.72	0.5
Genotype rs12252			
A/A	—	—	
A/G	0.80	0.44, 1.48	0.5
G/G	5.83	1.02, 45.7	**0** **.** **047**

Bold values indicate statistical significance (*p* < 0.05).

CI, confidence interval.

Among clinical factors, male sex remained a significant independent predictor of mortality [exp(*β*) = 2.30, 95% CI: 1.33–4.02; *p* = 0.003], as did immunodeficiency [exp(*β*) = 5.06, 95% CI: 1.28–24.6; *p* = 0.019]. The requirement for mechanical ventilation showed a strong association with mortality [exp(*β*) = 136, 95% CI: 30.8–1,232; *p* < 0.001]. However, the extremely wide confidence interval reflects the near-perfect separation between this variable and the outcome, and this estimate should be interpreted as a marker of severity rather than an independent causal effect. Age and length of stay remained significant after full adjustment (*p* = 0.048 and *p* < 0.001, respectively). Notably, ICU admission did not reach statistical significance in the Firth model [exp(*β*) = 4.43, 95% CI: 0.26–653; *p* = 0.3], likely due to collinearity with mechanical intubation, as virtually all patients requiring intubation were also admitted to the ICU. None of the remaining clinical variables, including BMI, Charlson Score, hypertension, diabetes, obesity, renal failure, COPD, or cancer, reached statistical significance in the fully adjusted model. All results are shown in Table 2.

### 3.3 Genetic associations with in-hospital mortality

The Firth penalized logistic regression model identified three SNP genotypes with nominally significant exploratory associations with in-hospital mortality. The rs2241880 G/G genotype (*ATG16L1*) was independently associated with increased mortality odds [exp(*β*) = 2.266; 95% CI: 1.090–4.870; *p* = 0.028], as was the rs12252 G/G genotype [*IFITM3*; exp(*β*) = 5.828; 95% CI: 1.024–45.7; *p* = 0.047]. The rs1800450 T/T genotype (*MBL2*) showed a significant protective association [exp(*β*) = 0.026; 95% CI: 0.000–0.945; *p* = 0.047]. No significant association was observed for any rs10754558 (*NLRP3*) genotype (all *p* > 0.4) (Table 2).

To evaluate the potential influence of mechanical ventilation as an intermediate variable in the causal pathway between genetic susceptibility and mortality, a complementary model was fitted excluding intubation status as a covariate. The rs1800450 T/T (*MBL2*) protective association remained statistically significant in both models (*p* = 0.047 with ventilation and *p* = 0.041 without), suggesting that its effect on mortality is independent of baseline disease severity as captured by ventilation status. The rs2241880 G/G (*ATG16L1*) association showed a directionally consistent effect in both models, though borderline in the model without mechanical ventilation [exp(*β*) = 1.84; *p* = 0.063]. In contrast, the rs12252 G/G (*IFITM3*) association did not retain nominal significance after removal of mechanical ventilation [exp(*β*) = 1.836; *p* = 0.385], suggesting that its effect may be partly mediated through disease severity rather than representing a direct independent effect on mortality. This is biologically plausible given that IFITM3 dysfunction may facilitate greater viral replication and more severe clinical progression (Brass et al. 2009). Complete results for both models, including Bonferroni and FDR-corrected *p*-values for all eight genotype comparisons, are provided in Supplementary Table S3.

As a sensitivity analysis, Bonferroni and Benjamini-Hochberg FDR corrections were applied across the eight genotype comparisons in both models. None of the associations retained statistical significance after correction in either model (all p_Bonf_ > 0.32; all p_FDR_ > 0.12), further supporting the hypothesis-generating nature of these findings and the need for replication in larger, independently powered cohorts.

To further contextualize these genetic findings, adjusted predictive probabilities were calculated for each genotype from the primary Firth model, holding all clinical covariates at their mean values (Figure 3). Among rs2241880 (*ATG16L1*) carriers, the adjusted probability of in-hospital death increased progressively across genotypes: 54.2% for A/A (reference), 57.5% for A/G, and 64.5% for G/G (*p* = 0.028 vs. A/A). For rs12252 (*IFITM3*), the G/G genotype showed an adjusted mortality probability of 75.5% compared to 58.0% for the A/A reference group (*p* = 0.047), a difference of 17.5 percentage points. For rs1800450 (*MBL2*), the T/T genotype showed a strikingly high adjusted survival probability of 88.7% compared to 40.7% for C/C carriers (*p* = 0.047); the biological plausibility and limitations of this unexpected finding are discussed in Section 4. No meaningful differences in adjusted probabilities were observed across rs10754558 (*NLRP3*) genotypes (all *p* > 0.4).

**Figure 3:**
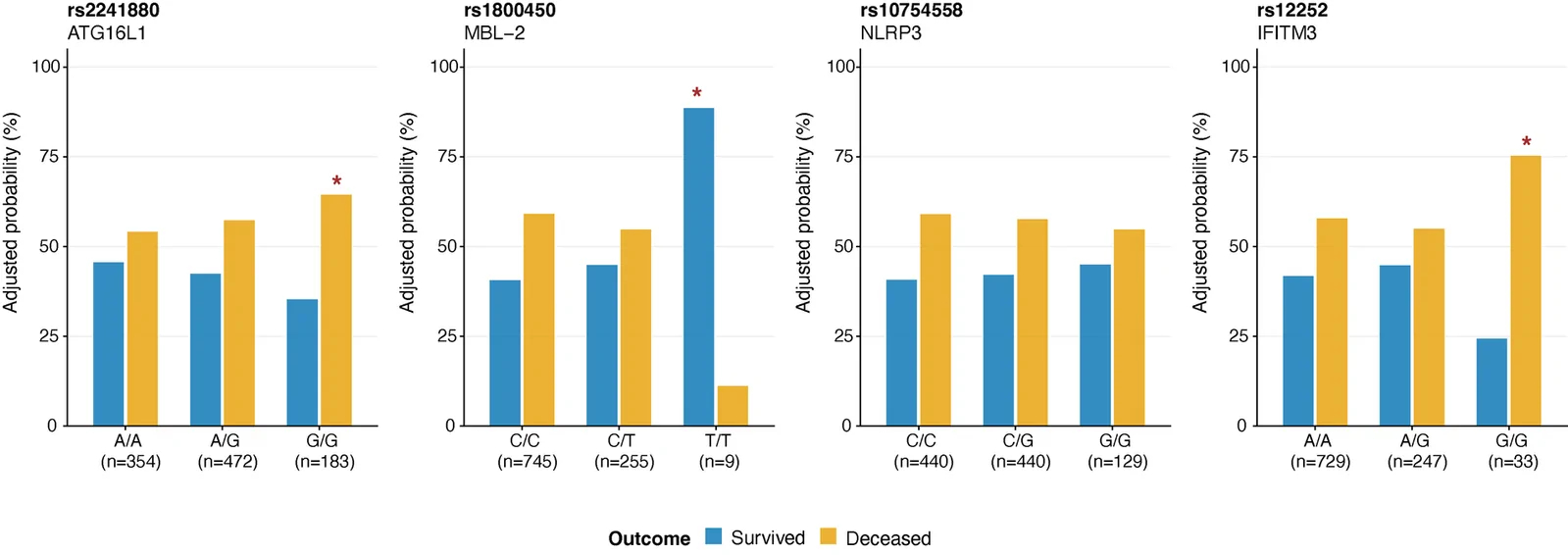
Adjusted predictive probabilities of in-hospital survival and mortality by SNP genotype. Bars represent the adjusted probability (%) of surviving (blue) or dying (orange) within each genotype group, derived as marginal effects from the Firth penalized logistic regression model, with all clinical covariates held at their mean values. Reference genotypes are shown for each SNP (rs2241880 A/A; rs1800450 C/C; rs10754558 C/C; rs12252 A/A). Asterisks indicate statistically significant differences compared to the reference genotype (*p < 0.05). Estimates for rs1800450 T/T (*n* = 9) and rs12252 G/G (*n* = 33) should be interpreted with caution due to small subgroup sizes and wide confidence intervals. N values indicate the total number of patients per genotype with available data.

Finally, given the significant ethnic diversity of the study cohort and the observed differences in allele frequencies across ethnic groups—particularly for rs12252 (*IFITM3*), whose variant G allele was more frequent in Black patients (21.6%) than in White (13.9%) or mixed race patients (16.3%) (*p* < 0.001)—an additional analysis was performed including self-reported ethnicity as a covariate in the Firth penalized logistic regression model (Supplementary Table S4). Patients with unknown ethnicity (*n* = 24) were excluded, resulting in a sensitivity model of *N* = 1,016. After the ethnicity adjustment, the odds ratios for the three SNPs of interest remained directionally consistent and of similar magnitude to those observed in the primary model: rs2241880 G/G [exp(*β*) = 2.03 vs. 2.27], rs12252 G/G [exp(*β*) = 5.37 vs. 5.83], and rs1800450 T/T [exp(*β*) = 0.03 vs. 0.03]. However, these associations did not reach statistical significance in the adjusted model (*p* = 0.060, *p* = 0.072, and *p* = 0.062, respectively), likely reflecting the modest reduction in sample size rather than confounding by ethnic background. Self-reported ethnicity itself was not independently associated with in-hospital mortality in this model (all *p* > 0.5).

### 3.4 Targeted variant analysis of candidate SNPs in HGI summary statistics

A two-stage targeted analysis was performed using the HGI R7 summary statistics. In the first stage, the pre-filtered HGI Release 7 files (*p* < 1 × 10^−5^) were interrogated, and none of the four candidate SNPs were identified, indicating that they did not reach suggestive significance in the global meta-analysis. In the second stage, complete regional data (±500 kb) were extracted from the full HGI R7 dataset using *tabix*, which confirmed that all four candidate SNPs are present in the complete dataset. None reached suggestive (*p* < 1 × 10^−5^) or genome-wide significance (*p* < 5 × 10^−8^) in any of the three phenotypes evaluated. In the hospitalized vs. non-hospitalized COVID-19 phenotype (B1), the observed *p*-values were: rs2241880 (*ATG16L1*) *p* = 0.206, rs1800450 (*MBL2*) *p* = 0.715, rs10754558 (*NLRP3*) *p* = 0.416, and rs12252 (*IFITM3*) *p* = 0.922. In the hospitalized COVID-19 vs. general population phenotype (B2), rs2241880 *p* = 0.694, rs1800450 *p* = 0.228, rs10754558 *p* = 0.603, and rs12252 *p* = 0.110. In the very severe COVID-19 vs. general population phenotype (A2), rs2241880 *p* = 0.678, rs1800450 *p* = 0.599, rs10754558 *p* = 0.769, and rs12252 *p* = 0.527. Complete association statistics including effect sizes and allele frequencies across all three phenotypes are provided in Supplementary Table S5. The proxy SNP analysis additionally identified at least one proxy with *R*^2^ = 1.0 for each candidate SNP across all three reference populations (European, African, and Admixed American), confirming that the candidate variants are well-tagged across diverse ancestral groups. However, the majority of the best proxies were not directly genotyped in the HGI R7 dataset, suggesting that these loci are sub-represented in the HGI contributing studies. Proxies that were present showed no significant association in any phenotype. Examination of all SNPs within the ±500 kb windows surrounding each candidate locus confirmed the absence of any regional association signal reaching suggestive significance across all three phenotypes, as illustrated in the local Manhattan plots (Supplementary Figure S2).

## 4 Discussion

This study investigated the relationship between SNPs in four genes associated with different aspects of innate immunity and COVID-19 in-hospital mortality in a cohort of 1,040 hospitalized Brazilian patients. The principal findings were threefold. First, the two SNPs rs2241880 G/G (*ATG16L1*) and rs12252 G/G (*IFITM3*) showed nominally significant exploratory associations with increased in-hospital mortality after full multivariate adjustment, though none survived correction for multiple testing. Second, rs1800450 T/T (MBL-2) showed a nominally significant exploratory protective association, an unexpected finding that warrants cautious interpretation given the small number of T/T carriers (*n* = 9). Third, clinical severity markers, particularly the requirement for mechanical ventilation, and demographic factors, including male sex and older age, were the primary independent predictors of fatal outcomes.

The clinical profile of non-survivors in our cohort was consistent with the established epidemiology of severe COVID-19. Older age, male sex, and higher comorbidity burden were significantly associated with fatal outcomes in univariate analysis, in line with numerous large-scale studies conducted during the pandemic (3, 24, 25). In the fully adjusted Firth model, male sex independently doubled the odds of mortality [exp(*β*) = 2.30; *p* = 0.003], a finding that has been attributed to sex-based differences in immune regulation, including in men a higher baseline inflammatory tone and lower innate antiviral responses, such as the release of type I IFNs (26–28). Immunodeficiency was also identified as a significant independent predictor of mortality [exp(*β*) = 5.06; *p* = 0.019], consistent with the known vulnerability of immunocompromised patients to severe SARS-CoV-2 infection and impaired viral clearance (29–31). Notably, the extremely wide confidence interval for this estimate (95% CI: 1.28–24.6) reflects the limited number of immunodeficient patients with complete data in this cohort (*n* = 26 with available records out of 593 total), and this finding should be interpreted cautiously.

The requirement for mechanical ventilation showed the strongest association with mortality in the Firth model [exp(*β*) = 136; *p* < 0.001]. This estimate should not be interpreted as an independent causal effect but rather as a consequence of the near-perfect separation between intubation and outcome, a statistical phenomenon that motivated the use of Firth's penalized regression (20, 21). Obesity was more prevalent among survivors than non-survivors (18% vs. 10%; *p* = 0.001), consistent with other studies where critically ill COVID-19 patients, in which higher BMI has been paradoxically associated with improved survival (32, 33). However, it is well documented that obesity is a risk factor for a higher rate of ICU admission and severe disease, compared to non-obese patients (23, 34).

In our cohort, the rs2241880 G/G genotype (*ATG16L1*) showed a nominally significant exploratory association with increased in-hospital mortality [exp(*β*) = 2.27; *p* = 0.028], with adjusted mortality probabilities of 64.5% in G/G carriers compared to 54.2% in the A/A reference group. This finding is consistent with the biological role of *ATG16L1* in autophagy, a cellular degradation process that constitutes a critical antiviral defense mechanism against SARS-CoV-2 (13, 35, 36). In the context of SARS-CoV-2 infection, impaired autophagy may limit lysosomal degradation of viral particles as well as antigen presentation, potentially contributing to viral persistence and a dysregulated inflammatory response. Supporting this hypothesis, autophagy-deficient models have demonstrated increased susceptibility to coronavirus-induced cytokine storms (37, 38). The consistency of this association across both the GLM (OR = 1.87; *p* = 0.036) and the Firth model [exp(*β*) = 2.27; *p* = 0.028] strengthens confidence in this finding.

The rs12252 G/G genotype (*IFITM3*) was associated with elevated mortality odds in the Firth model [exp(*β*) = 5.83; *p* = 0.047], with adjusted mortality probabilities of 75.5% among G/G carriers vs. 58.0% in A/A homozygotes. *IFITM3* encodes interferon-induced transmembrane protein 3, a restriction factor that inhibits viral membrane fusion and entry into host cells (39). The rs12252 G variant produces a truncated protein with reduced antiviral activity and has been previously associated with severe influenza outcomes and increased COVID-19 mortality across multiple populations (40–42). Notably, the association of rs12252 G/G with mortality did not retain nominal significance after removal of mechanical ventilation from the model [exp(*β*) = 1.836; *p* = 0.385], suggesting that its effect may be partly mediated through disease severity rather than representing a direct independent effect on mortality. This is biologically plausible, given that IFITM3 dysfunction may facilitate greater viral replication and more severe clinical progression, pathways that ultimately manifest as requirement for mechanical ventilation (40–42).

In our cohort, the variant G allele of rs12252 showed a substantially higher frequency in Black individuals (21.6%) compared to White (13.9%) and mixed patients (16.3%; *p* < 0.001). This is consistent with previously reported population-level differences in *IFITM3* allele frequencies across ancestral groups. This ethnic variability in allele frequency underscores the importance of characterizing these variants in admixed populations such as the Brazilian cohort studied here, where the overall G allele frequency (15.4%) falls between estimates reported for European and African ancestry populations (43). Nevertheless, the small number of G/G homozygotes in our sample (*n* = 34) yields a wide confidence interval (95% CI: 1.02–45.7), and this result must be considered exploratory and requires independent replication in larger cohorts.

We also observed that the rs1800450 T/T genotype (*MBL-2*), also known as the BB genotype, showed a significant association with reduced in-hospital mortality in the Firth model [exp(*β*) = 0.03; *p* = 0.047], with an adjusted survival probability of 88.7% among T/T carriers compared to 40.7% in C/C homozygotes. This direction of effect contradicts previous reports associating MBL deficiency with increased COVID-19 hospitalization and severity (14, 15), and must be interpreted with considerable caution given the very small number of T/T carriers in this cohort (*n* = 9). This finding is seemingly paradoxical since the T/T genotype is classically associated with reduced serum MBL levels and impaired complement lectin pathway activation, linked to increased susceptibility to various infectious diseases and, in some studies, more severe outcomes (15, 44). A possible explanation for this apparent contradiction may lie in the dual and context-dependent role of the complement lectin pathway during SARS-CoV-2 infection. While MBL-mediated complement activation is protective in the early, pre-inflammatory phase of infection by facilitating viral opsonization and clearance (45), excessive lectin pathway activation in the context of established critical illness may paradoxically amplify tissue injury through complement-mediated inflammation, endothelial damage, thrombosis, and higher IL-6 and TNF-α levels. All of these are hallmarks of severe COVID-19 pathophysiology (46–49). Under this framework, reduced MBL production in T/T carriers could attenuate complement-driven immunopathology in the most severe clinical stage, potentially conferring a survival advantage. Consistent with this interpretation, the T allele of rs1800450 has also been associated with protection against leprosy in a Brazilian population, suggesting that reduced MBL activity may confer a survival advantage in certain high pro-inflammatory infectious contexts in admixed Brazilian populations (50). Nonetheless, with only 9 T/T patients in our cohort, the confidence interval is extremely wide (95% CI: 0.00–0.94), rendering any definitive conclusion premature. This finding should be regarded as hypothesis-generating and warrants investigation in substantially larger cohorts with stratified analysis by disease phase.

Although the association of rs10754558 (*NLRP3*) with COVID-19 has been explored in several studies, no significant association was observed between rs10754558 (*NLRP3*) genotypes and in-hospital mortality in either multivariate model in this study. While the *NLRP3* inflammasome has been implicated in the cytokine storm associated with severe COVID-19—driving the maturation and secretion of IL-1β and IL-18—the rs10754558 variant in the 3' untranslated region may have a more subtle regulatory effect on inflammasome activation that is not detectable at the mortality endpoint in a cohort of this size (10, 11). Future studies incorporating longitudinal cytokine profiling alongside genotyping may be better positioned to elucidate the role of this polymorphism in COVID-19 pathophysiology.

It is important to acknowledge that racial and ethnic differences in COVID-19 outcomes have been documented in large-scale Brazilian cohorts. Zeiser et al. (51), analyzing 678,235 hospitalized patients during the first and second waves of the pandemic in Brazil, reported that while White patients represented the most prevalent hospitalized group, Black and mixed race patients exhibited higher in-hospital mortality rates. This pattern is consistent with the distribution observed in the present cohort, where mixed race patients showed a higher proportion of deaths (56.0%) compared to White (46.0%) and Black (45.7%) patients. These disparities are likely driven, at least in part, by structural socioeconomic inequalities, including differential access to healthcare, higher prevalence of comorbidities, and greater exposure to occupational risk, in addition to possible biological or genetic factors (51).

Independent replication of candidate gene associations in external datasets like the COVID-19 Host Genetics Initiative (HGI) is critical for validating genetic associations. However, a critical limitation of the HGI and of global genomic databases more broadly is the substantial underrepresentation of admixed Latin American populations. Brazil, despite boasting the world's largest recently admixed population, remains notably underrepresented in genomic research, with a substantial proportion of its genetic diversity absent from major public databases (52). This gap is highly consequential for COVID-19 research. Recent studies in Brazilian cohorts identified novel disease severity loci and rare variants systematically missed by Euro-centric analyses (53, 54).

In this context, we evaluated the four candidate SNPs in the HGI R7 summary statistics across three phenotypes of increasing clinical specificity: hospitalized vs. non-hospitalized COVID-19 (B1), hospitalized vs. general population (B2), and very severe COVID-19 vs. general population (A2). None of the four candidate SNPs reached suggestive (*p* < 1 × 10^−5^) or genome-wide significance (*p* < 5 × 10^−8^) in any of the three phenotypes analysed. We hypothecise that this lack of replication likely stems from two major factors: differences in clinical endpoints (our specific focus on in-hospital mortality vs. HGI's general hospitalization metrics) and distinct allele frequencies between the admixed Brazilian population and the predominantly European HGI cohorts. Thus, the results reported here should be interpreted as hypothesis-generating. Future multi-ancestry studies with matched mortality endpoints are essential to confirm these genetic contributions to COVID-19 outcomes in the Brazilian population.

Several limitations of this study should be acknowledged. First, the single-center design limits the generalizability of the findings to other healthcare settings and outpatient populations. Nonetheless, both survivors and non-survivors were drawn from the same source population of hospitalized COVID-19 patients, minimizing indication bias, and the multivariate models adjusted for the major clinical determinants of survival. Second, the small number of patients carrying rare genotypes, particularly rs1800450 T/T (*n* = 9) and rs12252 G/G (*n* = 34), substantially limits statistical power and yields wide confidence intervals that preclude definitive conclusions. Also, missing data for several comorbidities, most notably immunodeficiency (57% missing) and transplant status (73% missing), may have introduced residual confounding despite complete-case analysis. Standard acute physiological scoring systems recorded at admission, such as the Sequential Organ Failure Assessment (SOFA) or APACHE II scores, were not routinely available in our retrospective dataset. However, we mitigated this limitation by comprehensively adjusting for critical clinical proxies of acute disease severity—including ICU admission, mechanical ventilation, length of stay, and the Charlson Comorbidity Index—in our multivariate models. While these variables capture major manifestations of critical illness, residual confounding from acute organ dysfunction cannot be entirely ruled out. The interpretation of ethnic differences in allele frequencies warrants particular caution in the context of this study. Ethnicity was self-declared by patients according to IBGE categories, which captures socially constructed racial identity rather than genetic ancestry. This distinction is of considerable relevance in Brazil, whose population is characterized by one of the highest degrees of admixture globally, resulting from centuries of intermingling among European, African, and Indigenous ancestries (55). This includes the population of São Paulo (56). As a consequence, self-reported ethnicity may imperfectly reflect underlying genetic background, and allele frequency differences across ethnic groups may not fully capture true ancestral variation in these loci (57). To partially address this limitation, a sensitivity analysis adjusting for self-reported ethnicity was performed as a covariate in the Firth penalized logistic regression model. The genetic associations remained directionally consistent after this adjustment, as detailed in Supplementary Table S4 and Section 3.3.

Furthermore, the Asian subgroup comprised only six patients, precluding any meaningful interpretation of allele frequency estimates for this group. Formal correction for genetic ancestry using principal component analysis was not performed, as only four SNPs were genotyped, precluding the derivation of ancestry-informative components. Finally, the panel of four SNPs, chosen as representatives of four distinct aspects of innate immunity, represents only a small fraction of the possible genetic variation that may influence COVID-19 outcomes. None of the genetic associations survived Bonferroni or FDR correction, reinforcing the need for independent replication in larger cohorts. A genome-wide approach would be required to fully characterize the genetic architecture of disease severity in this population. Additionally, interaction analyses between SNP genotypes and clinical covariates such as age and sex were not performed in the present study, as the small sample sizes of rare genotype subgroups precluded statistically reliable estimation of interaction terms. Such analyses are warranted in future larger, multi-center cohorts with sufficient genotypic representation across all subgroups.

## 5 Conclusions

In this cohort of hospitalized COVID-19 patients in Brazil, in-hospital mortality was primarily determined by acute illness severity and demographic factors. Additional independent contributions from genetic variation in *ATG16L1* (rs2241880 G/G) and *IFITM3* (rs12252 G/G), implicates impaired autophagy and antiviral restriction as biologically plausible mechanisms of increased mortality risk. The protective effect on mortality associated with *MBL-2* rs1800450 T/T was unexpected and warrants further investigation in the context of complement-mediated immunopathology. External validation of these associations in publicly available large-scale GWAS summary statistics did not yield significant replication signals, consistent with the hypothesis-generating nature of these findings and the underrepresentation of admixed Brazilian populations in global genomic datasets. These findings contribute to the growing evidence supporting the role of host genetic factors in COVID-19 outcomes and should be regarded as hypothesis-generating, given the borderline significance levels and wide confidence intervals observed for some associations. Independent replication in larger, multi-center cohorts with diverse ethnic representation and comprehensive genetic profiling is needed to validate these associations and inform personalized risk stratification strategies.

## Data Availability

The original contributions presented in the study are included in the article and its Supplementary Material. A de-identified metadata spreadsheet containing the demographic and clinical variables, in-hospital outcome and genotypes for the four SNPs analysed, with all patient identifiers removed, is provided as Supplementary Data Sheet 1. Genotyping was performed by TaqMan real-time PCR allelic discrimination for four previously described variants catalogued in dbSNP (rs2241880, rs1800450, rs10754558, rs12252); no sequencing data were generated in this study. The HGI Release 7 summary statistics used in the targeted variant analysis are publicly available at https://www.covid19hg.org/results/r7/. Further inquiries can be directed to the corresponding author.
