# Supplementary material for: Exploratory analysis of innate immune gene polymorphisms and COVID-19 in-hospital mortality in a large admixed Brazilian cohort
Source: Front Med (Lausanne). 2026 Oct 7;13:1919908. doi: 10.3389/fmed.2026.1919908 (PMC13683161; doi:10.3389/fmed.2026.1919908)
Supplement: Supplementary file 1 [file Supplementaryfile1.docx]

Supplementary Material

# 1 Supplementary Tables

**Supplementary Table S1.** Allele frequencies of four candidate SNPs in the total cohort, stratified by in-hospital mortality outcome (N = 1,040) and ethnic group (N = 1,016).

| **Group** | **N** | **Genotype counts  (ref / het / var)** | **p (ref allele)** | **q (variant allele)** | **p-value** |
| --- | --- | --- | --- | --- | --- |
| **rs2241880 (ATG16L1) Reference allele: A Variant allele: G** |  |  | **p (A)** | **q (G)** | **p-value** |
| Total | 1040 | A/A:363 / A/G:490 / G/G:187 | 58.5% | 41.5% |  |
| Survived | 535 | A/A:186 / A/G:259 / G/G:90 | 59.0% | 41.0% |  |
| Deceased | 505 | A/A:177 / A/G:231 / G/G:97 | 57.9% | 42.1% | 0.659 |
| White | 656 | A/A:209 / A/G:314 / G/G:133 | 55.8% | 44.2% |  |
| Black | 81 | A/A:36 / A/G:35 / G/G:10 | 66.0% | 34.0% |  |
| Mixed (Pardo) | 273 | A/A:105 / A/G:127 / G/G:41 | 61.7% | 38.3% |  |
| Asian | 6 | A/A:5 / A/G:1 / G/G:0 | 91.7% | 8.3% | **0.001** |
| **rs1800450 (MBL-2)**  **Reference allele: C Variant allele: T** |  |  | **p (C)** | **q (T)** | **p-value** |
| Total | 1040 | C/C:765 / C/T:266 / T/T:9 | 86.3% | 13.7% |  |
| Survived | 535 | C/C:400 / C/T:128 / T/T:7 | 86.7% | 13.3% |  |
| Deceased | 505 | C/C:365 / C/T:138 / T/T:2 | 85.9% | 14.1% | 0.646 |
| White | 656 | C/C:474 / C/T:175 / T/T:7 | 85.6% | 14.4% |  |
| Black | 81 | C/C:68 / C/T:12 / T/T:1 | 91.4% | 8.6% |  |
| Mixed (Pardo) | 273 | C/C:202 / C/T:70 / T/T:1 | 86.8% | 13.2% |  |
| Asian | 6 | C/C:2 / C/T:4 / T/T:0 | 66.7% | 33.3% | **0.044** |
| **rs10754558 (NLRP3) Reference allele: C Variant allele: G** |  |  | **p (C)** | **q (G)** | **p-value** |
| Total | 1040 | C/C:456 / C/G:452 / G/G:132 | 65.6% | 34.4% |  |
| Survived | 535 | C/C:238 / C/G:224 / G/G:73 | 65.4% | 34.6% |  |
| Deceased | 505 | C/C:218 / C/G:228 / G/G:59 | 65.7% | 34.3% | 0.914 |
| White | 656 | C/C:271 / C/G:290 / G/G:95 | 63.4% | 36.6% |  |
| Black | 81 | C/C:42 / C/G:31 / G/G:8 | 71.0% | 29.0% |  |
| Mixed (Pardo) | 273 | C/C:133 / C/G:116 / G/G:24 | 70.0% | 30.0% |  |
| Asian | 6 | C/C:3 / C/G:3 / G/G:0 | 75.0% | 25.0% | **0.019** |
| **rs12252 (IFITM3) Reference allele: A Variant allele: G** |  |  | **p (A)** | **q (G)** | **p-value** |
| Total | 1040 | A/A:753 / A/G:253 / G/G:34 | 84.6% | 15.4% |  |
| Survived | 535 | A/A:396 / A/G:124 / G/G:15 | 85.6% | 14.4% |  |
| Deceased | 505 | A/A:357 / A/G:129 / G/G:19 | 83.5% | 16.5% | 0.197 |
| White | 656 | A/A:492 / A/G:145 / G/G:19 | 86.1% | 13.9% |  |
| Black | 81 | A/A:51 / A/G:25 / G/G:5 | 78.4% | 21.6% |  |
| Mixed (Pardo) | 273 | A/A:192 / A/G:73 / G/G:8 | 83.7% | 16.3% |  |
| Asian | 6 | A/A:1 / A/G:3 / G/G:2 | 41.7% | 58.3% | **<0.001** |

p (ref allele) = frequency of the reference allele; q (variant allele) = frequency of the variant allele. Genotype counts presented as: homozygous reference (ref), heterozygous (het), homozygous variant (var). p-value (Survived vs. Deceased): Pearson's chi-squared test comparing allele frequencies between outcome groups. p-value (across ethnic groups): Pearson's chi-squared test comparing allele frequencies across White, Black, Mixed/Pardo, and Asian groups. The Asian group comprises only 6 patients and should be interpreted with caution. 24 patients with unknown ethnicity are included in the Total, Survived, and Deceased groups but excluded from ethnic group-specific analyses.

**Supplementary Table S2.** Multivariate logistic regression analysis of independent predictors of in-hospital mortality (standard GLM, excluding acute severity markers).

| **Characteristic** | **OR** | **95% CI** | **p-value** |
| --- | --- | --- | --- |
| **Age** | 1.04 | 1.01, 1.06 | **0.002** |
| **Sex** |  |  |  |
| Female | — | — |  |
| Male | 2.04 | 1.32, 3.18 | **0.001** |
| **BMI** | 0.99 | 0.97, 1.00 | 0.10 |
| **Charlson score** | 1.12 | 0.93, 1.37 | 0.2 |
| **Length of Stay** | 0.98 | 0.97, 1.0 | **0.006** |
| **Hypertension** |  |  |  |
| No | — | — |  |
| Yes | 0.87 | 0.53, 1.41 | 0.6 |
| **Diabetes** |  |  |  |
| No | — | — |  |
| Yes | 1.12 | 0.67, 1.86 | 0.7 |
| **Obesity** |  |  |  |
| No | — | — |  |
| Yes | 0.86 | 0.46, 1.59 | 0.6 |
| **Renal Failure** |  |  |  |
| No | — | — |  |
| Yes | 0.97 | 0.41, 2.37 | >0.9 |
| **Immunodeficiency** |  |  |  |
| No | — | — |  |
| Yes | 3.21 | 1.05, 11.4 | 0.051 |
| **COPD** |  |  |  |
| No | — | — |  |
| Yes | 0.67 | 0.22, 2.16 | 0.5 |
| **Cancer** |  |  |  |
| No | — | — |  |
| Yes | 1.81 | 0.57, 6.54 | 0.3 |
| **Genotype rs2241880** |  |  |  |
| A/A | — | — |  |
| A/G | 1.09 | 0.68, 1.76 | 0.7 |
| G/G | 1.87 | 1.05, 3.40 | **0.036** |
| **Genotype rs1800450** |  |  |  |
| C/C | — | — |  |
| C/T | 0.84 | 0.52, 1.36 | 0.5 |
| T/T | 0.00† |  | >0.9 |
| **Genotype rs10754558** |  |  |  |
| C/C | — | — |  |
| C/G | 0.98 | 0.62, 1.54 | >0.9 |
| G/G | 0.68 | 0.35, 1.34 | 0.3 |
| **Genotype rs12252** |  |  |  |
| A/A | — | — |  |
| A/G | 0.85 | 0.53, 1.39 | 0.5 |
| G/G | 1.48 | 0.47, 5.40 | 0.5 |
| CI = Confidence Interval, OR = Odds Ratio. †Complete separation occurred for rs1800450 T/T in the standard GLM; the estimate is numerically unstable and should not be interpreted. Firth penalized regression was used as the primary model to address this issue (Supplementary Table S3). | | | |

**Supplementary Table S3.** Comparison of Firth penalized logistic regression models with and without mechanical ventilation as a covariate.

| **SNP** | **Genotype** | **Model with mechanical ventilation** | | | | **Model without mechanical ventilation** | | | |
| --- | --- | --- | --- | --- | --- | --- | --- | --- | --- |
|  |  | **exp(β)** | **p-value** | **p Bonferroni** | **p FDR** | **exp(β)** | **p-value** | **p Bonferroni** | **p FDR** |
| ATG16L1 rs2241880 | A/G | 1.269 | 0.440 | 1.000 | 0.546 | 1.110 | 0.699 | 1.000 | 0.699 |
| ATG16L1 rs2241880 | G/G | 2.266 | 0.028* | 0.226 | 0.125 | 1.838 | 0.063 | 0.504 | 0.252 |
| MBL2 rs1800450 | C/T | 0.722 | 0.287 | 1.000 | 0.546 | 0.743 | 0.269 | 1.000 | 0.604 |
| MBL2 rs1800450 | T/T | 0.026 | 0.047* | 0.375 | 0.125 | 0.057 | 0.041* | 0.328 | 0.252 |
| NLRP3 rs10754558 | C/G | 0.902 | 0.719 | 1.000 | 0.719 | 0.867 | 0.575 | 1.000 | 0.658 |
| NLRP3 rs10754558 | G/G | 0.729 | 0.466 | 1.000 | 0.546 | 0.676 | 0.302 | 1.000 | 0.604 |
| IFITM3 rs12252 | A/G | 0.803 | 0.478 | 1.000 | 0.546 | 0.850 | 0.550 | 1.000 | 0.658 |
| IFITM3 rs12252 | G/G | 5.828 | 0.047* | 0.373 | 0.125 | 1.836 | 0.385 | 1.000 | 0.616 |

***** p < 0.05 (nominal significance). exp(β), exponentiated regression coefficient (odds ratio); p Bonferroni, Bonferroni-corrected p-value (threshold p < 0.0063 for 8 comparisons); p FDR, Benjamini-Hochberg false discovery rate-adjusted p-value (threshold p < 0.05). Reference genotypes: rs2241880 A/A, rs1800450 C/C, rs10754558 C/C, rs12252 A/A. Both models include the following covariates: age, sex, BMI at admission, Charlson comorbidity score, length of hospital stay, ICU admission, hypertension, diabetes, obesity, renal failure, immunodeficiency, COPD, and cancer. The model without mechanical ventilation excludes intubation status as a covariate. All models were fitted using Firth penalized logistic regression (*logistf* package, R).

**Supplementary Table S4.** Firth penalized logistic regression with ethnicity adjustment (N = 1,016).

| **Characteristic** | **exp(β)** | **95% CI** | **p-value** |
| --- | --- | --- | --- |
| **Age** | 1.03 | 1.00, 1.06 | 0.051 |
| **Sex** |  |  |  |
| Female | — | — |  |
| Male | 2.26 | 1.29, 4.00 | **0.004** |
| **BMI at Admission** | 0.99 | 0.97, 1.01 | 0.2 |
| **Charlson Score** | 1.10 | 0.86, 1.41 | 0.4 |
| **Length of Stay** | 0.94 | 0.93, 0.96 | **<0.001** |
| **Requirement for ICU** |  |  |  |
| No | — | — |  |
| Yes | 4.30 | 0.25, 634 | 0.3 |
| **Intubation Requirement** |  |  |  |
| No | — | — |  |
| Yes | 144 | 31.9, 1,359 | **<0.001** |
| **Hypertension** |  |  |  |
| No | — | — |  |
| Yes | 0.90 | 0.47, 1.70 | 0.7 |
| **Diabetes** |  |  |  |
| No | — | — |  |
| Yes | 1.05 | 0.55, 2.03 | 0.9 |
| **Obesity** |  |  |  |
| No | — | — |  |
| Yes | 0.61 | 0.28, 1.33 | 0.2 |
| **Renal Failure** |  |  |  |
| No | — | — |  |
| Yes | 1.89 | 0.58, 6.79 | 0.3 |
| **Immunodeficiency** |  |  |  |
| No | — | — |  |
| Yes | 5.35 | 1.35, 26.5 | **0.016** |
| **COPD** |  |  |  |
| No | — | — |  |
| Yes | 0.42 | 0.12, 1.63 | 0.2 |
| **Cancer** |  |  |  |
| No | — | — |  |
| Yes | 1.28 | 0.32, 6.30 | 0.7 |
| **Ethnicity** |  |  |  |
| White | — | — |  |
| Black | 1.16 | 0.42, 3.47 | 0.8 |
| Mixed (Pardo) | 1.14 | 0.64, 2.06 | 0.6 |
| Other | 0.25 | 0.01, 45.9 | 0.5 |
| **Genotype rs2241880 (ATG16L1)** |  |  |  |
| A/A | — | — |  |
| A/G | 1.27 | 0.68, 2.36 | 0.5 |
| G/G | 2.03 | 0.97, 4.37 | 0.060 |
| **Genotype rs1800450 (MBL-2)** |  |  |  |
| C/C | — | — |  |
| C/T | 0.70 | 0.38, 1.30 | 0.3 |
| T/T | 0.03 | 0.00, 1.19 | 0.062 |
| **Genotype rs10754558 (NLRP3)** |  |  |  |
| C/C | — | — |  |
| C/G | 0.94 | 0.53, 1.67 | 0.8 |
| G/G | 0.74 | 0.32, 1.78 | 0.5 |
| **Genotype rs12252 (IFITM3)** |  |  |  |
| A/A | — | — |  |
| A/G | 0.82 | 0.44, 1.52 | 0.5 |
| G/G | 5.37 | 0.87, 44.0 | 0.072 |
| exp(β) = exponentiated regression coefficient (odds ratio); CI = confidence interval. Patients with unknown ethnicity were excluded, resulting in N = 1,016. Bold p-values: p < 0.05. | | | |

**Supplementary Table S5.** Association statistics for candidate SNPs in the HGI (R7) summary statistics across three COVID-19 severity phenotypes.

| **Gene** | **Candidate SNP** | **Phenotype** | **CHR** | **POS (GRCh38)** | **REF** | **ALT** | **Beta** | **SE** | **p-value** | **AF (ALT)** | **N cases** | **N controls** |
| --- | --- | --- | --- | --- | --- | --- | --- | --- | --- | --- | --- | --- |
| *ATG16L1* | rs2241880 | B1 | 2 | 233,274,722 | A | G | 0.02195 | 0.01737 | 0.206 | 0.4829 | 16,512 | 71,321 |
| *ATG16L1* | rs2241880 | B2 | 2 | 233,274,722 | A | G | 0.00327 | 0.00831 | 0.694 | 0.4829 | 44,052 | 2,350,426 |
| *ATG16L1* | rs2241880 | A2 | 2 | 233,274,722 | A | G | -0.00521 | 0.01258 | 0.678 | 0.4949 | 17,864 | 1,139,586 |
| *MBL2* | rs1800450 | B1 | 10 | 52,771,475 | C | T | 0.00900 | 0.02468 | 0.715 | 0.1350 | 16,512 | 71,321 |
| *MBL2* | rs1800450 | B2 | 10 | 52,771,475 | C | T | 0.01435 | 0.01191 | 0.228 | 0.1350 | 44,627 | 2,355,515 |
| *MBL2* | rs1800450 | A2 | 10 | 52,771,475 | C | T | 0.00952 | 0.01811 | 0.599 | 0.1379 | 17,928 | 1,144,541 |
| *NLRP3* | rs10754558 | B1 | 1 | 247,448,734 | G | C | -0.01680 | 0.02066 | 0.416 | 0.5675 | 9,823 | 66,362 |
| *NLRP3* | rs10754558 | B2 | 1 | 247,448,734 | G | C | 0.00466 | 0.00895 | 0.603 | 0.5675 | 37,841 | 2,278,250 |
| *NLRP3* | rs10754558 | A2 | 1 | 247,448,734 | G | C | 0.00390 | 0.01330 | 0.769 | 0.5803 | 16,911 | 1,064,258 |
| *IFITM3* | rs12252 | B1 | 11 | 320,772 | A | G | -0.00342 | 0.03502 | 0.922 | 0.0753 | 16,227 | 69,997 |
| *IFITM3* | rs12252 | B2 | 11 | 320,772 | A | G | -0.02633 | 0.01646 | 0.110 | 0.0753 | 43,347 | 2,010,480 |
| *IFITM3* | rs12252 | A2 | 11 | 320,772 | A | G | 0.01724 | 0.02726 | 0.527 | 0.0584 | 17,436 | 1,074,944 |

Beta, effect size on log-odds scale for the ALT allele from inverse-variance weighted meta-analysis; SE, standard error; AF, allele frequency of the ALT allele; B1, hospitalized COVID-19 vs. non-hospitalized COVID-19 (16,512 cases / 71,321 controls); B2, hospitalized COVID-19 vs. general population (44,986 cases / 2,356,386 controls); A2, very severe respiratory COVID-19 vs. general population (18,152 cases / 1,145,546 controls); CHR, chromosome; POS, genomic position (GRCh38 build); REF, reference allele; ALT, alternative allele. HGI R7, COVID-19 Host Genetics Initiative Release 7 (April 2022).

# 2 Supplementary Figures

**Supplementary Figure S1. Ethnic composition and distribution of variant allele frequencies in the study cohort.** **(A)** Overall ethnic composition among patients with available ethnicity data (n = 1,016). **(B)** Proportion of survivors and deceased within each ethnic group; p-value from Pearson's chi-squared test (p = 0.012). **(C)** Frequency of the variant allele (q) for each of the four candidate SNPs across ethnic groups. p-values indicate chi-squared tests comparing allele frequencies across all four groups. Variant alleles: rs2241880 G; rs1800450 T; rs10754558 G; rs12252 G. Ethnicity groups reported here are self-reported. Complete allele frequency data are presented in Supplementary Table S1.

**Supplementary Figure S2. Local Manhattan plots of candidate SNP loci in the HGI Release 7 multi-ancestry summary statistics.** Local Manhattan plots (±500 kb) centered at the genomic position of each candidate SNP across three HGI Release 7 phenotypes: hospitalized COVID-19 versus non-hospitalized COVID-19 (B1), hospitalized COVID-19 versus general population (B2), and very severe respiratory COVID-19 versus general population (A2). Each plot displays all SNPs within a ±500 kb window around the candidate SNP position on the x-axis (chromosomal position in megabases) and the −log₁₀(p-value) on the y-axis. The candidate SNP is highlighted as a red diamond. Horizontal dashed lines indicate genome-wide significance (p < 5×10⁻⁸, red) and suggestive significance (p < 1×10⁻⁵, orange). Points are colored by distance to the candidate SNP: dark blue (< 100 kb), light blue (100–250 kb), and very light blue (> 250 kb). All analyses were performed using the HGI R7 multi-ancestry meta-analysis summary statistics (ALL ancestry, excluding 23andMe; GRCh38 build).
